# Learning from cardiogenic shock deaths: a comparative analysis between hypotensive and normotensive cardiogenic shock

**DOI:** 10.1093/ehjopen/oeaf053

**Published:** 2025-06-18

**Authors:** Patrick Tran, Mithilesh Joshi, Prithwish Banerjee, Sendhil Balasubramanian, Uday Dandekar, Emmanuel Otabor, Stephen Adeyeye, Jaffar Al-Sheikhli, Michael Kuehl

**Affiliations:** Centre for Health and Life Sciences, Coventry University, Priority St., Coventry, CV1 5FB, UK; University Hospitals Coventry & Warwickshire, Clifford Bridge Road, Coventry, CV2 2DX, UK; Centre for Health and Life Sciences, Coventry University, Priority St., Coventry, CV1 5FB, UK; University of Warwick, Medical School Building, Coventry, CV4 7AL, UK; Centre for Health and Life Sciences, Coventry University, Priority St., Coventry, CV1 5FB, UK; University Hospitals Coventry & Warwickshire, Clifford Bridge Road, Coventry, CV2 2DX, UK; University of Warwick, Medical School Building, Coventry, CV4 7AL, UK; University Hospitals Coventry & Warwickshire, Clifford Bridge Road, Coventry, CV2 2DX, UK; University Hospitals Coventry & Warwickshire, Clifford Bridge Road, Coventry, CV2 2DX, UK; Centre for Health and Life Sciences, Coventry University, Priority St., Coventry, CV1 5FB, UK; Centre for Health and Life Sciences, Coventry University, Priority St., Coventry, CV1 5FB, UK; Centre for Health and Life Sciences, Coventry University, Priority St., Coventry, CV1 5FB, UK; University of Warwick, Medical School Building, Coventry, CV4 7AL, UK; Centre for Health and Life Sciences, Coventry University, Priority St., Coventry, CV1 5FB, UK; University of Warwick, Medical School Building, Coventry, CV4 7AL, UK

**Keywords:** Cardiogenic shock, Normotensive cardiogenic shock, Mortality, Heart failure

## Abstract

**Aims:**

This study characterizes the under-recognized normotensive cardiogenic shock (CS) phenotype by analysing fatal cases, comparing haemodynamics, shock trajectories, and management gaps with hypotensive CS.

**Methods and results:**

We analysed 112 patients who died from CS between 2017 and 2022. Patients > 70 were excluded due to local eligibility criteria. Normotensive (*n* = 51) and hypotensive CS (*n* = 61) had similar degrees of cardiac impairment, with cardiac indices well below 2.0 L/min/m^2^ and LVEF < 35%. Both groups exhibited comparable end-organ dysfunction, including lactate levels (7.0 ± 5.0 vs. 6.1 ± 5.6 mmol/L, *P* = 0.441) and acute liver injury (51–56%). Hypotensive CS typically followed a predictable decline in shock stage [75.4% deteriorated to Society for Cardiovascular Angiography Interventions (SCAI) stages D–E], whereas normotensive CS demonstrated less predictable trajectories, with 51% showing apparent stability before rapid deterioration and death. Receiver operating characteristic analysis revealed that only the rise in serum creatinine weakly predicted deterioration to advanced SCAI stages (Area under the curve 0.62, *P* = 0.035), while initial lactate and liver function tests lacked predictive value. Normotensive cases had a median 14 h longer referral window from onset of CS but were referred less frequently. Twenty-six were considered potential candidates for advanced heart failure therapy but were not referred.

**Conclusion:**

Normotensive and hypotensive CS share similar degrees of hypoperfusion but differ in their shock trajectories. The delay in referrals for normotensive CS highlights the need for earlier recognition of this phenotype and standardized protocols to ensure timely referrals for mechanical circulatory support.

## Introduction

Cardiogenic shock (CS) is defined by end-organ hypoperfusion due to cardiac dysfunction, regardless of blood pressure.^1^ Yet, most clinical trials have historically emphasized systolic blood pressure (SBP) < 90 mmHg as a defining threshold, overlooking patients with normotensive CS, who maintain SBP through compensatory vasoconstriction despite profound hypoperfusion.^2^ These patients often face higher mortality rates than hypotensive patients with preserved perfusion.^3^ While variations in treatment responses between acute myocardial infarction (AMI) and heart failure (HF) CS are well recognized, distinctions between hypotensive and normotensive CS remain poorly understood—a critical gap highlighted by international experts.^1,4^

Despite the DanGer Shock trial demonstrating improved outcomes with early mechanical circulatory support (MCS), CS mortality remains high at 40–50%.^5^ The trial emphasized learning from mortality cases, not just survivors. Accordingly, we analysed CS-related deaths to compare shock trajectories and perfusion profiles in normotensive vs. hypotensive CS. Using the Society for Cardiovascular Angiography Interventions (SCAI) five-stage classification, we examined shock evolution from admission to 12–24 h before death to identify patterns of decline that may inform earlier referrals for MCS.

## Methods

### Study design and setting

This retrospective study analysed CS mortality cases (2017–2022) at a UK tertiary centre with 24/7 primary percutaneous coronary intervention but limited to intra-aortic balloon pump as its only MCS. The nearest advanced HF centre (AHFC) is located 30 miles away.

### Study population

**Figure 1:**
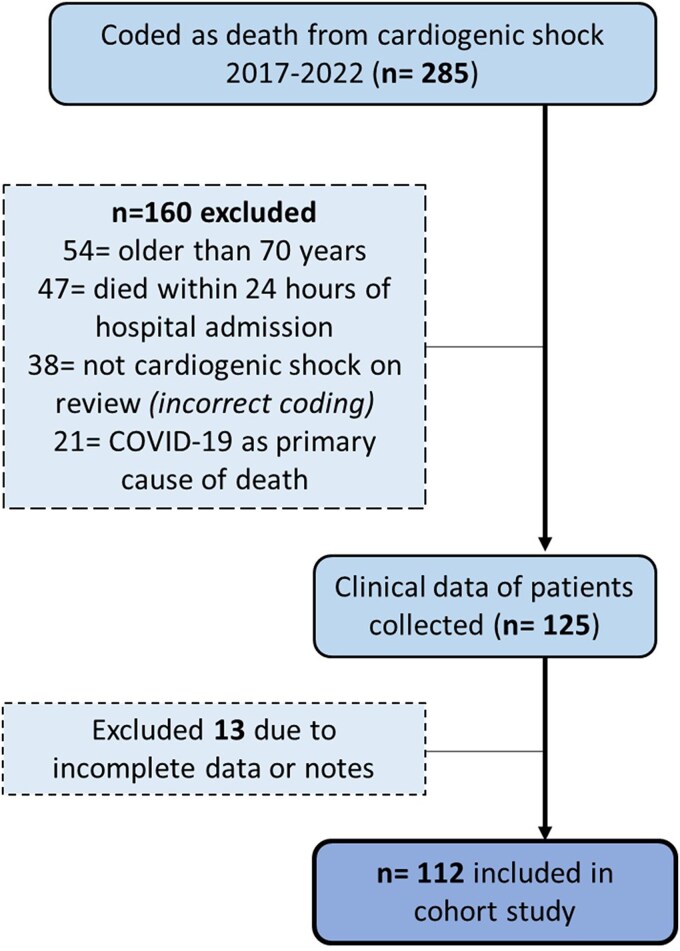
Flow diagram of patients included and excluded.

We included patients aged <70 years who died of CS, surviving >12 h post-shock onset. This age cut-off aligns with regional criteria for transplant eligibility and MCS candidacy. Cases were identified from the British Cardiovascular Intervention Society audit data set, Hospital Episodes Statistics, and Clinical Coding records. Of the 285 screened patients, 112 patients met inclusion criteria (*Figure 1*).

### Definition of cardiogenic shock and study groups

Cardiogenic shock was defined using a combination of clinical, biochemical, and haemodynamic evidence of end-organ hypoperfusion, corresponding to the SCAI stages C–E criteria. For hypotensive CS, patients were required to demonstrate both cardiac dysfunction [e.g. cardiac index < 2.2 L/min/m^2^ estimated on echocardiogram, or left ventricular ejection fraction (LVEF) < 35%] and sustained SBP < 90 mmHg, or the use of vasoactive support to maintain SBP ≥ 90 mmHg, plus clinical and biochemical features of tissue hypoperfusion (e.g. cool extremities, oliguria < 0.5 mL/kg/h, lactate > 2.5 mmol/L, rising serum creatinine, elevated bilirubin, or transaminase).

Normotensive CS was defined as impaired cardiac function with tissue hypoperfusion (above criteria) and SBP > 90 mmHg, before any vasoactive therapy. Alternative shock aetiologies (e.g. sepsis or obstructive) were excluded. To ensure clear separation between normotensive and hypotensive cohort and to confirm that normotensive CS cases did not rapidly progress to hypotension, SBP measurements were averaged over 4–6 h following CS onset (4–6 readings) before any vasopressor initiation. This approach prevented misclassification of early hypotensive CS as normotensive CS, ensuring the two represented distinct phenotypes.

### Statistical analysis

Continuous variables were presented as mean ± SD or median with interquartile range (IQR) and compared using t-test or Mann–Whiney U test, as appropriate. Categorical data were analysed with χ^2^ or Fisher’s exact test. Receiver operating characteristic (ROC) analysis identified any predictors of SCAI shock deterioration. A *P* < 0.05 was considered significant. Analyses were performed using SPSS v26 and GraphPad Prism v9.

## Results

### Normotensive vs. hypotensive cardiogenic shock profile

*Table 1* summarizes the baseline characteristics and CS phenotype. Normotensive CS patients had markedly higher SBP (117 ± 18 vs. 77 ± 12 mmHg) yet showed comparable hypoperfusion to its hypotensive counterpart, including severe lactic acidosis (median lactate ≥ 6 mmol/L) and acute liver injury (51–56%). Hypertension was more common in the normotensive group (88.2% vs. 32.8%, *P* < 0.001). Both groups had similar degrees cardiac impairment by LVEF and estimated cardiac index, well below 2.0 L/min/m^2^.

**Table 1:** Comparison between normotensive vs. hypotensive cardiogenic shock phenotypes

Baseline characteristics	CS with systolic BP < 90mmHg (*n* = 61)	CS with systolic BP ≥ 90mmHg (*n* = 51)	*P* value
Age, mean ± SD	60.4 ± 8.2	58.9 ± 9.9	0.365
Range, years	30–69	26–70	–
Male, *n* (%)	45 (73.8)	37 (72.5)	0.884
Charlson Comorbidity Index	4 (3–5)	5 (2–6)	0.425
eGFR (mL/min/1.73 m^2^), mean ± SD	82.1 ± 40.3	84.9 ± 31.4	0.688
Known DNAR order, *n* (%)	2 (3.3)	1 (2.0)	0.667
SCAI grade on admission, *n* (%)			
At risk	10 (18.4)	19 (37.3)	0.034
Beginning of shock	17 (27.9)	16 (31.4)	
Classical cardiogenic shock	13 (21.3)	8 (15.7)	
Deteriorating	4 (6.6)	0 (0)	
Extremis (e.g. cardiac arrest)	17 (27.9)	8 (15.7)	
Cardiogenic shock phenotype			
OOH cardiac arrest, *n* (%)	11 (18.0)	11 (21.6)	0.643
Blood pressure, mean ± SD			
Systolic	77.4 ± 11.4	117.1 ± 21.8	<0.001
Diastolic	51.4 ± 11.4	68.8 ± 15.1	
Mean arterial pressure	59.6 ± 9.9	85.0 ± 15.2	
Average heart rate, mean ± SD	93.8 ± 10	107.2 ± 15	<0.001
Oliguria (<0.5 mL/kg/h), *n* (%)	39 (63.9)	27 (52.9)	0.254
Pulmonary oedema, *n* (%)	32 (52.5)	26 (51.0)	0.876
LVEF, mean ± SD	29.7 ± 15.6	34.7 ± 16.1	0.135
Echo-derived cardiac index, mean ± SD^a^	1.68 ± 0.52	1.85 ± 0.66	0.420
Missing data (no LVOT VTI available)	8	10	–
RV systolic dysfunction, *n* (%)	15 (31.3)	14 (34.1)	0.823
Not reported RV function, *n* (%)	11	8	–
Lactate, mean ± SD	7.0 ± 5.0	6.1 ± 5.6	0.441
SCr rise from baseline, mean ± SD	92.7 ± 95.6	78.8 ± 115.9	0.508
Bilirubin			
Rise > 25 µmol/L, *n* (%)	14 (23.0)	11 (21.6)	0.861
Bilirubin, median (IQR)	17 (7–24)	17 (9–40)	0.839
ALT			
Rise >50 µmol/L, *n* (%)	34 (55.7)	26 (51.0)	0.615
ALT, median (IQR)	101 (38 -504)	84 (35–378)	0.517
Organ support during hospital stay			
ITU input, *n* (%)	45 (73.8)	32 (62.7)	0.322
Decision for palliation	7 (11.5)	11 (21.6)	
Primary PCI, *n* (%)	18 (29.5)	16 (31.4)	0.831
IABP	12 (19.7)	4 (7.8)	0.104
Noradrenaline	16 (26.2)	11 (21.6)	
Adrenaline	6 (9.8)	1 (2.0)	0.118
Dobutamine	8 (13.1)	11 (21.6)	
Metaraminol	5 (8.2)	5 (9.8)	
Milrinone	2 (3.3)	2 (3.9)	
Vasopressin	2 (3.3)	0 (0)	
Discussion with local MCS centre	7 (11.5)	3 (5.9)	0.501
Not applicable as palliated on ITU	11 (18)	12 (23.5)	
Mortality data			
Aetiology of CS, *n* (%)			0.616
Acute myocardial infarction	38 (62.3)	30 (58.8)	
Decompensated heart failure	20 (32.8)	17 (33.3)	
Acute myocarditis	2 (3.3)	1 (2.0)	
Isolated right heart failure	1 (1.6)	2 (3.9)	
Constrictive pericarditis	0	1 (2)	
Concurrent sepsis, *n* (%)	7 (11.5)	7 (13.7)	0.779
Anoxic brain injury, *n* (%)	19 (31.1)	17 (33.3)	0.841
LOHS, median (IQR)	9 (4–17)	5 (2–12)	0.003
Duration of CS from onset to death/WLST, median (IQR), hours	20 (14–40)	34 (18–62)	0.037

^a^Cardiac index (L/min/m^2^) was estimated echocardiographically as a product of heart rate and stroke volume (estimated by the left ventricular outflow tract cross-sectional area and velocity time integral using the continuity equation, divided by body surface area).

ALT, alanine transaminase; eGFR, estimated glomerular filtration rate; IABP, intra-aortic balloon pump; ITU, intensive therapy unit; LOHS, length of hospital stay; LVEF, left ventricular ejection fraction; LVOT VTI: left ventricular outflow tract velocity time integral; OOH, out-of-hospital; PCI, percutaneous coronary intervention; RV, right ventricle; SCAI, Society for Cardiovascular Angiography and Interventions; SCr, serum creatinine (µmol/L); WLST, withdrawal of life-sustaining treatment.

### Society for Cardiovascular Angiography Interventions shock trajectory

**Figure 2:**
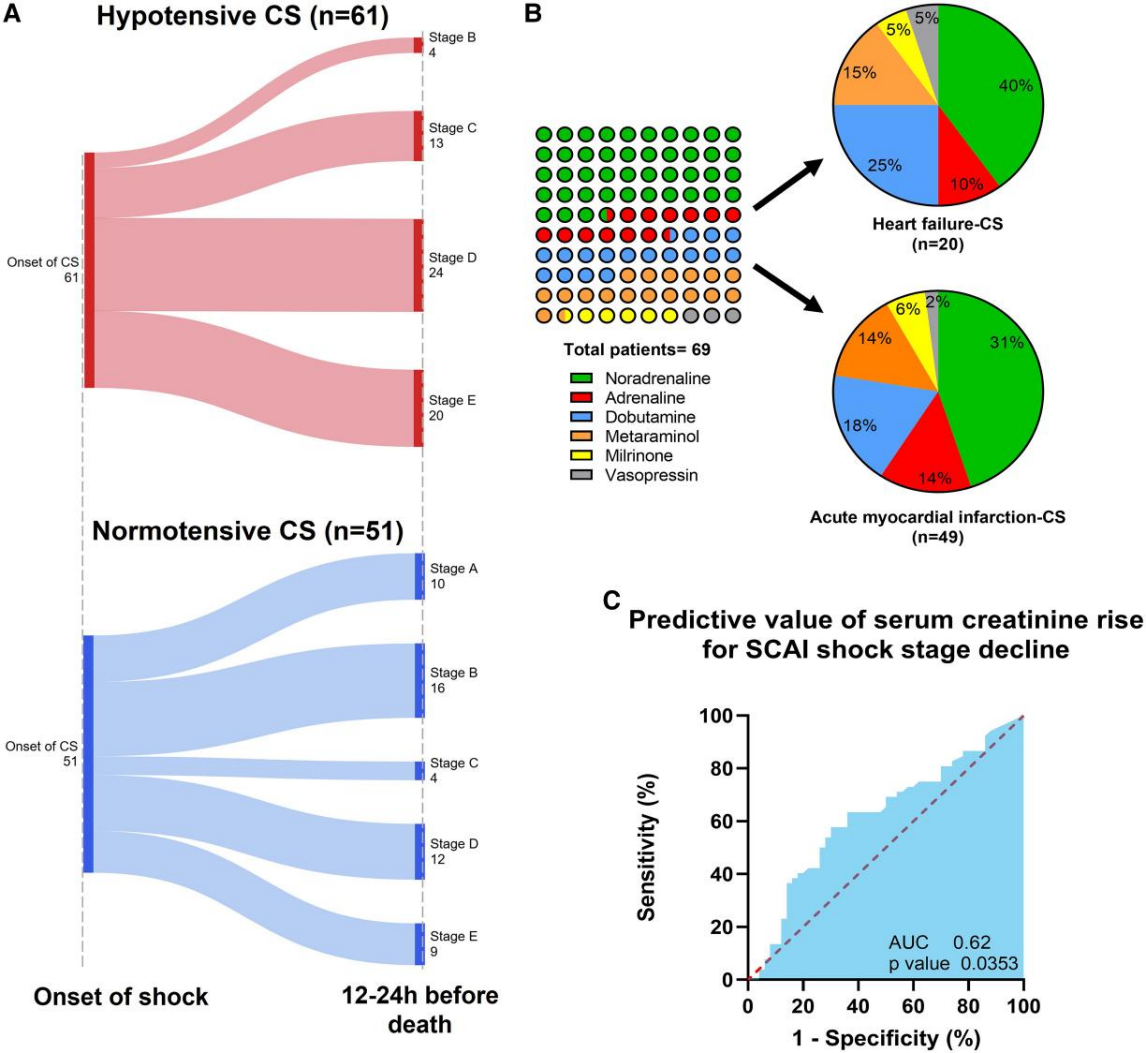
(*A*) Sankey diagram illustrating the progression of Society for Cardiovascular Angiography Interventions shock stage from cardiogenic shock onset to 12–24 h before death in hypotensive and normotensive groups; (*B*) initial choice of inotrope/vasopressor agent; (*C*) receiver operating characteristic analysis of the predictive value in serum creatinine rise for forecasting a worsening shock trajectory.

Shock evolution differed significantly (*Figure 2*). While 75.4% of hypotensive CS progressed to SCAI stage D/E, normotensive CS patients showed initial improvement in 51% before subsequent deterioration, with a longer median time to death (34 vs. 20 h, *P* = 0.037). Of all biomarkers assessed, only acute kidney injury (median serum creatinine rise +77.5 vs. +39.0 µmol/L, *P* = 0.035) weakly predicted SCAI shock deterioration, while initial lactate (6.8 ± 6.0 vs. 6.4 ± 4.4 mmol/L, *P* = 0.682) and liver function test lacked predictive value for shock trajectory.

### Inotropic and vasopressor use

Noradrenaline was the most commonly used agent in both groups (*Table 1*), including for both AMI-CS and non-AMI-CS (*Figure 2*). While there was no statistically significant difference in the distribution of inopressors, dobutamine use was higher in normotensive CS, and adrenaline was more frequent in hypotensive CS.

### Referrals to advanced heart failure centre

Only 10 patients were discussed with the AHFC, all of whom were declined due to clinical instability and delayed referral. Among the eligible patients, 26 (18 hypotensive and 8 normotensive) met criteria for short-term MCS, but were not referred. Median time from onset of normotensive CS to death was 34 h (IQR 9–62), 14 h longer than in the hypotensive group (20 h, IQR 6–40, *P* = 0.037), yet normotensive cases were less likely to be referred.

## Discussion

This study demonstrates that normotensive CS presents with a severity of hypoperfusion comparable to hypotensive CS with similar lactate elevations and end-organ dysfunction, challenging the assumption that normotensive CS is less severe. Despite maintaining a SBP > 90 mmHg for at least 4–6 h after CS onset, patients with normotensive CS exhibited equally depressed LVEF and echo-derived cardiac indices as those with hypotensive CS. However, while hypotensive CS typically followed predictable trajectory of clinical decline, normotensive CS often show misleading periods of apparent stability before rapid deterioration, creating a false reassurance for clinicians and delaying appropriate escalation.

Recent data show a rise in normotensive CS prevalence, increasing from 5.2% in the SHOCK registry to 12.1% in contemporary registries.^6^ Many of these patients had pre-existing hypertension, potentially allowing them to sustain SBPs > 90 mmHg despite significant drops in mean arterial pressure (MAP) and tissue hypoperfusion. This supports emerging evidence that microcirculatory flow, rather than BP alone, may be more predictive of outcomes.^7^

Although the SCAI shock classification provides a helpful framework, it has limitations in capturing CS complexity, offering only snapshots of clinical markers that may not align perfectly into single stages. It also fails to account for the temporal dynamics of CS, especially in normotensive cases where stages may improve yet remain at risk of rapid deterioration.^8^

Current treatment strategies reveal inconsistences in the use of inotropes and vasopressors. Noradrenaline was widely used in both AMI-CS and non-AMI-CS despite their different pathophysiology. In HF-related CS, maladaptive vasoconstriction and increased afterload can make vasopressor use harmful. We noted that metaraminol was used in normotensive CS to raise MAP and overcome tissue hypoperfusion, despite limited supporting evidence. Conversely, milrinone, in combination with low-dose noradrenaline, may offer haemodynamic advantages in HF-CS by reducing afterload, yet were underutilized in this cohort.^9^

Despite a longer median referral window, normotensive CS patients were less likely to be referred for advanced HF care, resulting in missed opportunities for MCS. The absence of hypotension often masked the true severity of shock, delaying recognition of the need for LV unloading to prevent the downward spiral of multi-organ failure. Protocols triggering a shock team activation, flagged up by early warning scores ≥ 3, have been proposed to improve timely referrals to AHFC.^4,10^

## Limitations

This study’s retrospective, single-centre design and exclusion of patients > 70 years old limit the generalizability of its findings. Selection bias may also be present due to the exclusion of patients who died within 24 h. Key cardiac biomarkers, e.g. troponin and natriuretic peptides, were unavailable in >20% patients and thus excluded in ROC analysis. Additionally, the absence of invasive haemodynamic monitoring including direct cardiac index measurements restricted the granularity of physiology assessment.

## Conclusion

Normotensive CS is associated with similar degrees of tissue hypoperfusion and cardiac dysfunction as hypotensive CS, but their shock trajectories differ. Management strategies must evolve to account for this phenotype, including earlier recognition, refined inotrope use, reconsideration of BP thresholds in CS classification, and serial SCAI staging to guide care.

## Data Availability

The data that support the findings of this study are available from the corresponding author upon reasonable request.

## Lead author biography

Dr Patrick Tran is a UK cardiology trainee with a developed interest in the biomechanics and pharmacotherapy of heart failure, device therapy, cardiorenal metabolic medicine and a generalist to the human body.
